# Sex-Linked Growth Disorder and Aberrant Pituitary Gene Expression in Nestin-Cre-Mediated *Egr1* Conditional Knockout Mice

**DOI:** 10.3390/biology12070966

**Published:** 2023-07-06

**Authors:** Cody Swilley, Yu Lin, Yuze Zheng, Xiguang Xu, Min Liu, Kurt Zimmerman, Hehuang Xie

**Affiliations:** 1 Epigenomics and Computational Biology Lab, Fralin Life Sciences Institute of Virginia Tech, Blacksburg, VA 24061, USA; codys20@vt.edu (C.S.); yulin96@vt.edu (Y.L.); yz5968@nyu.edu (Y.Z.); xiguang@vt.edu (X.X.); lmin73@vt.edu (M.L.); 2 Department of Biomedical Sciences and Pathobiology, Virginia-Maryland College of Veterinary Medicine, Virginia Tech, Blacksburg, VA 24061, USA; 3 Genetics, Bioinformatics and Computational Biology Program, Virginia Tech, Blacksburg, VA 24061, USA; 4 Translational Biology, Medicine and Health Program, Virginia Tech, Blacksburg, VA 24061, USA; 5 School of Neuroscience, Virginia Tech, Blacksburg, VA 24061, USA

**Keywords:** sex difference, *Egr1*, Nestin, RNA-seq, pituitary, growth

## Abstract

**Simple Summary:**

Reduced growth hormone levels were observed in mice without the *Egr1* gene and mice carrying a Nestin-Cre driver. It remains unknown why these two strains of mice share a similar phenotype and whether the dysregulation of hormone production in the pituitary occurs via the same mechanism. Previous studies have shown conflicting results regarding the effectiveness of Nestin-Cre in driving gene knockout in the pituitary. In this study, we found that while the Nestin-Cre driver successfully removed *Egr1* expression in neuronal lineage cells, it did not effectively remove it from all sections of the pituitary. Moreover, the Nestin-Cre driver alone caused growth abnormalities in the mice and influenced the expression of genes related to growth factors in the pituitary. Sex differences were also observed in the mice that exhibited these growth and gene expression abnormalities, with female mice being more sensitive to the presence of Nestin-Cre and the loss of *Egr1*. Overall, this study highlights the limitations of the Nestin-Cre driver in removing *Egr1* from pituitary cells and provides insights into the impact of Nestin-Cre on growth and gene expression, particularly in relation to sex differences.

**Abstract:**

Genes that regulate hormone release are essential for maintaining metabolism and energy balance. *Egr1* encodes a transcription factor that regulates hormone production and release, and a decreased in growth hormones has been reported in *Egr1* knockout mice. A reduction in growth hormones has also been observed in Nestin-Cre mice, a model frequently used to study the nervous system. Currently, it is unknown how *Egr1* loss or the Nestin-Cre driver disrupt pituitary gene expression. Here, we compared the growth curves and pituitary gene expression profiles of Nestin-Cre-mediated *Egr1* conditional knockout (*Egr1*cKO) mice with those of their controls. Reduced body weight was observed in both the Nestin-Cre and *Egr1*cKO mice, and the loss of *Egr1* had a slightly more severe impact on female mice than on male mice. RNA-seq data analyses revealed that the sex-related differences were amplified in the Nestin-Cre-mediated *Egr1* conditional knockout mice. Additionally, in the male mice, the influence of *Egr1*cKO on pituitary gene expression may be overridden by the Nestin-Cre driver. Differentially expressed genes associated with the Nestin-Cre driver were significantly enriched for genes related to growth factor activity and binding. Altogether, our results demonstrate that Nestin-Cre and the loss of *Egr1* in the neuronal cell lineage have distinct impacts on pituitary gene expression in a sex-specific manner.

## 1. Introduction

The pituitary gland is an organ that controls endocrine homeostasis by releasing a number of hormones to regulate growth, metabolism, stress responses, and the reproductive system. It can be divided into three sections: the anterior, intermediate, and posterior lobes [1]. Each section comprises distinct types of pituitary-specific cells characterized classically by the hormones they synthesize. Recent single-cell transcriptome profiling of the rat anterior pituitary identified folliculostellate cells, five hormone-producing cells, endothelial cells, and blood cells [2]. In the mouse pituitary, a single-cell analysis led to the identification of a “multi-hormonal” cell cluster that expressed growth hormone (GH), prolactin (PRL), and thyroid-stimulating hormone (TSH) simultaneously [3]. The well-characterized gonadotropes in the pituitary gland produce gonadotropins, luteinizing hormone (LH), and follicle-stimulating hormone (FSH). These hormones control gonadal function and development in both sexes. LH and FSH are heterodimeric glycoproteins that share a common α-glycoprotein hormone subunit but have distinct β subunits. The expression of the LH-β subunit (LH_β_) is regulated by three key transcription factors: steroidogenic factor 1 (*sf1*), pituitary homeobox factor 1 (*Pitx1*), and early growth response factor 1 (*Egr1*) [4,5,6,7,8].

The *Egr1* gene encodes a transcription factor with three zinc finger binding domains that recognize the consensus GC-rich genomic sequence 5′-GCG(T/G)GGGCG-3′, regardless of the methylation status of the cytosine [9]. As an immediate early gene, *Egr1* can be rapidly and transiently induced in response to various environmental stimuli [10,11,12]. Activation of the *Egr1* gene does not require de novo protein synthesis. During cell specification, the EGR1 protein has been found to serve as a critical epigenetic regulator that recruits a DNA demethylation enzyme to remove cytosine methylation for downstream gene activation [13]. In the anterior pituitary, *Egr1* induction via gonadotropin-releasing hormone (GnRH) plays a critical role in regulating the synthesis of LH_β_ in gonadotrope cells [4,14]. A recent single-cell analysis demonstrated that gonadotrope cells exposed to GnRH exhibit the concentration-dependent bimodal response pattern of *Egr1* expression [15]. Furthermore, as GnRH concentration increases, the proportion of *Egr1-*expressing cells increases accordingly. The functional role of *Egr1* in the pituitary was demonstrated in transgenic mice with complete knockout *(Egr1KO*) of the *Egr1* gene [16]. The loss of the *Egr1* gene led to a deficiency in LH_β_ production and female infertility. These *Egr1*KO mice were of a smaller stature than the control mice, and the size of the pituitary gland was reduced in both sexes. It was determined that the *Egr1* gene affects the cell differentiation of the somatotrophs and consequently results in a lower level of GH [17].

Many pituitary hormones have sex-specific functions and are synthesized in response to physiologic stimuli related to sex differences. A recent transcriptomic study revealed that significant sex differences in pituitary gene expression can be detected before puberty in mice; they can be detected as early as postnatal day 12 and persist post-puberty at least until postnatal day 37 [18]. Pituitary cellular composition can differ according to sex as well [3]. The percentage of gonadotropes in the male pituitary is approximately twice that in the female pituitary, and this difference is accompanied by a relative predominance of somatotropes over lactotrophs. In the female rat pituitary, the *Egr1* gene promoter and first intron provide cis-regulatory elements that drive the cell type-specific expression of *Egr1* in LH_β_ expressing cells [19,20]. In the male rat, *Egr1* is expressed in a subset of somatotrophs and lactotrophs, and it is co-expressed with LH_β_ in gonadotrophs. Still, its transcription was not observed in pituitary precursor cells expressing TSH, adrenocorticotropic hormone (ACTH), or Sox2 [21]. Despite the important role of *Egr1* in the development of gonadotropes, the male *Egr1*KO mice did not show reproduction defects as striking as those observed in the female mice. This sex difference was speculated to be associated with the dynamic regulation of *Egr1* expression in gonadotrophs during the estrous cycle [16,17]. The contribution of the *Egr1* gene to pituitary sexual dimorphism remains poorly understood.

The Nestin-Cre transgenic mouse is one of the mouse models most frequently used to study gene functions in the central nervous system. The Nestin gene encodes a member of the intermediate filament proteins, which are primary components of the cytoskeleton. It was initially discovered in neural stem cells, but has also been located in several different tissues throughout the body, including the pancreas, small bowel, muscles, and pituitary [22]. One of the first studies using Nestin-Cre mice targeted the glucocorticoid receptor and found the Nestin-Cre mice to be smaller than the control mice, and to exhibit lower anxiety responses [23]. Higher anxiety thresholds and smaller body sizes are consistently reported in Nestin-Cre transgenic mice [24]. This was speculated to be the result of the hypothalamic expression of the human growth hormone gene inserted downstream of the Cre recombinase. An early study on Nestin-Cre transgene expression found that it was present in the embryonic pituitary as well as in the stem cells of the adult pituitary [25]. However, it is still unknown whether it can achieve high efficiency in Nestin-Cre-mediated recombination in pituitary cells.

In this study, we utilized the Nestin-Cre driver to generate *Egr1* conditional knockout (*Egr1*cKO) mice without *Egr1* gene expression in their neuronal lineage cells. We monitored the growth rates and examined the sex-related differential gene expressions in the pituitary glands of the *Egr1*cKO mice, comparing them with those of the Nestin-Cre controls. Our aim was to gain insights into the potential interplay between the *Egr1* gene and the Nestin-Cre driver in the disruption of pituitary gene expression. Additionally, we sought to investigate the effectiveness of Nestin-Cre-mediated recombination in removing the *Egr1* gene specifically from pituitary cells.

## 2. Materials and Methods

### 2.1. Animals

All animal experiments were performed in accordance with the Virginia Tech (Blacksburg, VA, USA) Institutional Animal Care and Use Committee guidelines. The *Egr1* conditional knockout mouse strain (*Egr1*_tm1a_A04, C57BL/6N-*Egr1*<tm1a(NCOM)Mfgc>/Tcp; MGI:5766027) was purchased from the Centre for Phenogenomics, Canada. The Nestin-Cre (B6.Cg-*Tg (Nes-cre)1Kln*/J; Jackson Lab, #003771) was a kind gift from Dr. Michael Fox’s lab. To create the *Egr1*cKO mice, a breeding scheme was implemented to cross individuals with varying degrees of floxed *Egr1* alleles with heterozygous Nestin-Cre mice until the desired Nestin-Cre-driven *Egr1*cKO was achieved. The mice were maintained and bred in a 12 h light/dark cycle under standard pathogen-free conditions. Free food (Teklad Global 18% Protein Rodent Diet) and water access was provided.

### 2.2. Measurement of Growth

The weights of the *Egr1*cKO mice and their corresponding wild-type littermates, as well as those of the heterozygous Nestin-Cre positive mice and the controls, were measured for 12 weeks. Each data point represents the average of at least ten mice, and standard derivations (SDs) are indicated.

### 2.3. Genotyping PCR

The mice were genotyped at three weeks of age, during the time of weaning. A small distal portion of the tail was removed and incubated at 55 °C overnight in DirectPCR lysis reagent (Viagen, Cedar Park, TX, USA, cat# 101T) solution along with Proteinase K (Invitrogen, Carlsbad, CA, USA, cat# AM 2546). The next day, the sample was incubated at 80 °C for one hour to deactivate the proteinase K before preparing for PCR sampling. Genotyping PCR reactions were performed according to the Jackson Laboratory’s protocol for Nestin-Cre and the Centre for Phenogenomics’ protocol for *Egr1*-loxp. A GoTaq G2 Green Master Kit (Promega, Madison, WI, USA, cat# M7822) and *Egr1* primers (*Egr1*KO-GT-For:5′-GGG AGG GTT TGT TTT GAT GA-3′ and *Egr1*KO-GT-Rev: 5′-CCA GCA CCC TAG TGGCTA CA-3′) or Nestin-Cre primers (Nes-CRE SET R: 5′-TGC ATG ATC TCC GGT ATT GA-3′ and Nes-CRE SET- F 5′-CGT ACT GAC GGT GGG AGA AT-3′) were used to create an *Egr1* or Nestin-Cre PCR mixture. Agarose gel (General Purpose LE, El Cajon, CA, USA, Cat# 20-101) electrophoresis was used to identify the PCR products. A GeneRuler 100 bp DNA ladder (ThermoFisher, Waltham, MA, USA, Cat# SM0241) was used to determine the PCR product sizes.

### 2.4. Immunohistochemistry (IHC)

The *Egr1*cKO mice and the controls were rapidly and deeply anesthetized with isoflurane (Vet One, Boise, ID, USA, cat# 502017) and perfused transcardially with 10% formalin into the left ventricle. The right ventricle was opened to allow for exsanguination. After cardiac arrest was confirmed, the mice went through cervical dislocation. The brain was exposed and the maximum amount of bone was removed to allow for further tissue fixation in 10% formalin overnight. The pituitary was left in the sella turcica and dissected away from the sphenoid bone. It was then fixed for 48 h in 10% formalin before being removed. Both tissues, once fixed, were sent to the pathology laboratory of the Virginia–Maryland College of Veterinary Medicine for embedment. Coronal slices of the tissues in the wax block were taken and sent back to the pathology laboratory. Both the whole brain and the pituitary gland went through an automotive system involving a Ventana Discovery Ultra machine (DAB Detection kit Cat#: 760-159) and secondary antibody OMap anti-Rb HRP Cat#: 760-4311, purchased from Roche Diagnostic, Indianapolis, USA. Both the whole brain and the pituitary gland were stained with anti-*Egr1* rabbit antibody (41542, purchased from Cell Signaling Technology, Danvers, MA, USA). Images were acquired using a MoticEasy Scan Pro 6 slide scanner.

### 2.5. RNAscope

Additional tissue samples were taken from the slices of embedded tissue used for IHC. An RNAscope procedure was carried out in accordance with the Advanced Cell Diagnostics protocol for RNAscope^®^ Multiplex Fluorescent Reagent Kit v2 Assay (Document Number 323100-USM). Briefly, the FFPE sections were baked at 60 °C for 1 h in a dry oven, deparaffinized, and incubated with hydrogen peroxide at RT for 10 min. After target retrieval, the sections were incubated with probes and put through three sequential amplifications followed by signal development. Probes (Mm-*Egr1*-C3 REF 423371-C3, Mm-Lhb-C2 REF 478401-C2, and Mm-Gh REF 445361) were used for RNAscope detection. The slides were counterstained with DAPI and mounted with Prolong Gold anti-fade mountant (Invitrogen, cat# P36930). Images were taken using a Zeiss LSM 880 confocal microscope from the Virginia Tech Fralin Imaging Center.

### 2.6. RNA Extraction, RNA-Seq Library Construction, and Data Analysis

RNA was extracted from the pituitary using an RNaeasy Mini Kit (Qiagen, Hilden, Germany, Cat# 74104). The homogenized samples were subjected to the protocol provided by the manufacturer. The RNA concentrated on the silica membrane was eluted with RNase-free molecular biology-grade water. A total of 150 ng of RNA was collected from each tissue sample and shipped to Novogene Corporation Inc., Sacramento, CA, USA. for RNA-seq library construction. The libraries were sequenced on the Hiseq 4000 platform using the 150 bp paired-end mode (Illumina, San Diego, CA, USA). Trim Galore (version 0.6.5) was used to filter short or low-quality reads and trim adapter sequences from raw reads. Clean reads were mapped to the mm10 genome, and expression was quantified using STAR (version 2.7.3a). The raw counts were employed to pairwisely identify differentially expressed genes using R package DESeq2. Genes with a greater than 1.5-fold change and adjusted *p*-value of less than 0.05 were considered significant. R package glmmSeq was used to perform a mixed-model analysis and assess significant *Egr1*KO-dependent genes (adjusted *p*-value < 0.05).

### 2.7. GO Analysis

A gene ontology (GO) analysis was performed and visualized using R package clusterProfiler (v4.4.4). Default parameters were used for the biological process (BP) enrichment analysis. The resulting GO terms and corresponding *p*-values were then processed using R package rrvgo to reduce GO term redundancy.

### 2.8. Statistical Analysis

The data in the growth curves are expressed as mean ± SD. A Student’s *t*-test was used to determine the significant differences between the two groups with the following critical values: * for *p* < 0.05 and ** for *p* < 0.01.

## 3. Results

### 3.1. Generation and Characterization of Egr1cKO Mice

**Figure 1:**
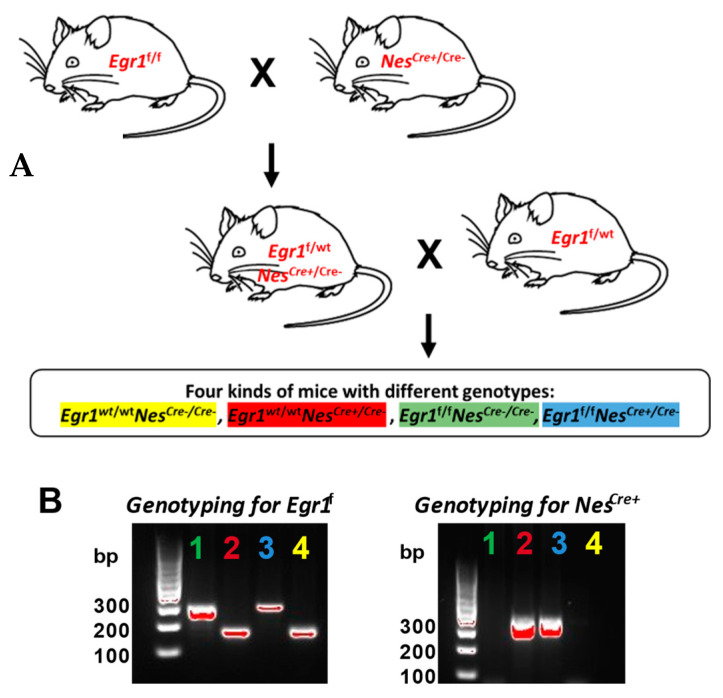
Generation of *Egr1*cKO mice. (**A**) Breeding scheme to obtain *Egr1*cKO mice. Four kinds of mice with different genotypes were included in this study, with *Egr1*^f/f^ Nes^Cre+^ being the desired knockout genotype and the remaining genotypes being used as controls. The desired knockout genotype and the controls are all color coded throughout the paper. *Egr1*^f^ represents a floxed *Egr1* allele and Nes^Cre+^ represents the presence of Nestin-Cre in the loxp system, while Nes^Cre−^ represents its absence. (**B**) Genotyping PCR to validate the presence of Nestin-Cre or *Egr1* in the loxp systems of the mice. *Egr1*^f^ is 291 bp while wild-type is 165 bp; Nes^Cre+^ is 350 bp while Nes^Cre−^ has no band.

Both Nestin-Cre and *Egr1*KO mice show impaired pituitary functions. To explore the commonalities and sex differences resulting from the Nestin-Cre driver and *Egr1* gene, a total of eight groups of mice with four different genotypes were included in this study (Figure 1A). Their genotypes are denoted as *Egr1*^f^Nes^cre+^, *Egr1*^f^Nes^cre−^ *Egr1*^wt^Nes^cre+^, and *Egr1*^wt^Nes^cre−^ in the text following Figure 1. Only heterozygous Nestin-Cre mice were used in this breeding scheme to ensure that only one copy of the Nestin-Cre driver would be present in the genome of the Nes^cre+^ mice, while the *Egr1*^f^ mice had two copies of *Egr1*-loxp to ensure the removal of the *Egr1* gene from the two paired chromosomes. We performed genotyping PCR reactions to determine the presence or absence of the Nestin-Cre driver and *Egr1*-loxp, respectively (Figure 1B). The Nes^cre+^ mice yielded a PCR product at 350 bp, while no PCR product could be obtained from the wild-type controls. Using primers provided by the Centre for Phenogenomics in Canada, PCR products with 102 bp and 378 bp were obtained from the homozygous *Egr1*^f^ mice and the wild-type controls, respectively.

### 3.2. Growth Reduction Observed in Both Nestin-Cre and Egr1cKO Mice

**Figure 2:**
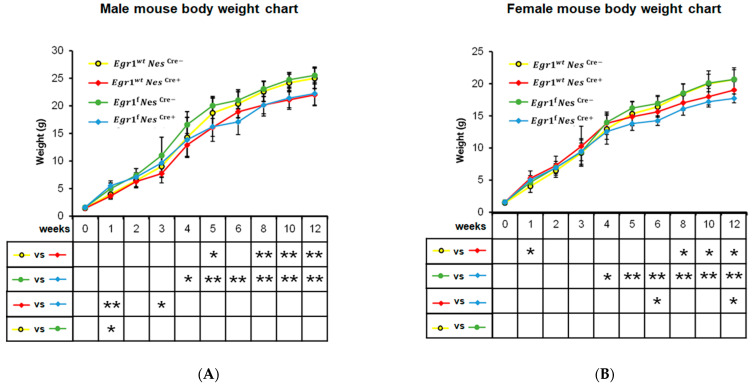
Growth curves for (**A**) male and (**B**) female mice of four genotypes from birth to 12 weeks. The data for each time point were derived from 10 individuals and are presented as means ± SEM. A group comparison was performed using a Student’s *t*-test. * denotes *p*-value < 0.05 and ** denotes *p*-value < 0.01.

Despite the known impact of the Nestin-Cre driver and the loss of the *Egr1* gene on body weight, no growth curve has been provided to demonstrate the progressive changes that occur in *Egr1*cKO and Nestin-Cre mice during postnatal development. Therefore, we monitored the eight groups of mice for twelve weeks after their birth (Figure 2). The difference the growth rate of the *Egr1*^wt^Nes^cre+^ and that of the *Egr1*^wt^Nes^cre−^ mice became significant with the approach of young adulthood and was consistent after eight weeks. Compared with the female mice, the males showed more significant differences, with a *p*-value ≤ 0.01 from 8 to 12 weeks. Compared with the controls of the same sex, reductions in body weight of around 12% and 8% were observed in the male and female *Egr1*^wt^Nes^cre+^ mice, respectively. At the twelfth week, the average weight of a male *Egr1*^wt^Nes^cre+^ mouse was 22.0 ± 1.98 g, while the average weight of a male *Egr1*^wt^Nes^cre−^ mouse was 25.0 ± 1.87 g. The average weight of a female *Egr1*^wt^Nes^cre+^ mouse was 19.0 ± 1.30 g, while the average weight of a female *Egr1*^wt^Nes^cre−^ mouse was 20.7 ± 1.82 g.

A previous study indicated that the body weights of both male and female homozygous *Egr1*KO mice were significantly and consistently lower than those of normal controls of the same sex at ages older than two weeks [17]. In our study, the conditional *Egr1* knockout delayed such differences between the sexes. Starting at four weeks of age, the *Egr1*^f^Nes^cre+^ and *Egr1*^f^Nes^cre−^ mice exhibited reduced body weight steadily and with increasing significance. In the twelfth week, the average weight of a male *Egr1*^f^Nes^cre+^ mouse was 22.2 ± 2.07 g, and the average weight of a male *Egr1*^f^Nes^cre−^ mouse was 25.5 ± 1.53 g, i.e., a greater than 12% body mass reduction in male *Egr1*cKO mice. Similarly, an approximately 14% reduction win body mass as observed for female *Egr1*cKO mice, which weighed on average 17.7 ± 0.68 g, while female *Egr1*^f^Nes^cre−^ mice weighed 20.7 ± 1.50 g on average. In the early weeks, some significant differences in weight were sporadically observed. This is likely due to technical variations being enlarged by the lightweight litter. Interestingly, compared with female *Egr1*^wt^Nes^cre+^ mice, slightly decreased body weights were observed in the female *Egr1*^f^Nes^cre+^ mice, with *p* values ≤ 0.05 at six and twelve weeks. This result suggests that the loss of *Egr1* might have a more noticeable impact on female mice than on male mice.

### 3.3. Sex Differences in Gene Expression Amplified in Nestin-Cre and Egr1cKO Mice

**Figure 3:**
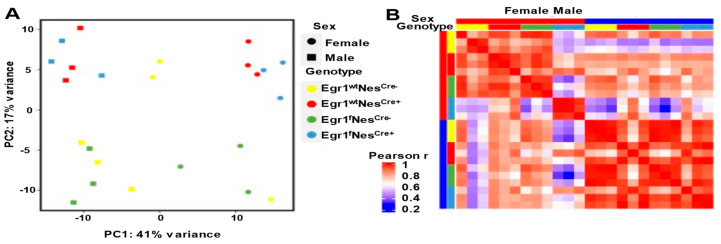
RNA-seq data clustering. (**A**) Principal component analysis (PCA) and (**B**) Pearson correlations of pituitary gland transcriptome data. RNA-seq analyses were performed in triplicates for each group, and all samples were from mice 12 weeks of age.

The growth differences observed in the Nestin-Cre and *Egr1*cKO mice inspired us to examine their pituitary gene expression profiles. RNA-seq analyses were performed in biological triplicates on adult pituitary tissues isolated from the eight groups of mice, with each sample taken at 12 weeks of age. A principal component analysis (PCA) indicated that, according to their gene expression patterns, the twenty-four samples could be segregated into two clusters, with females on the right side of the graph and males on the left (Figure 3A). Pearson correlation coefficients of the gene expressions across the samples further confirmed sex-specific clustering in general (Figure 3B). Intriguingly, strong correlations in the gene expressions in the pituitary tissues across all four genotypes were observed for males, but not for females. More specifically, despite a strong correlation among the other three biological replicates, the gene expression profiles from the female *Egr1*^f^Nes^cre+^ pituitary tissues showed a weak correlation (a Pearson’s r of around 0.2–0.4) with those of the Nes^cre−^ mice, but intermediate to strong correlations (Pearson’s rs of approximately 0.6–0.8) with those of both male and female Nes^cre+^ mice. This result suggests that gene expression differences resulting from different genotypes (Nes^cre+^ in particular) may prevail over sex differences.

**Figure 4:**
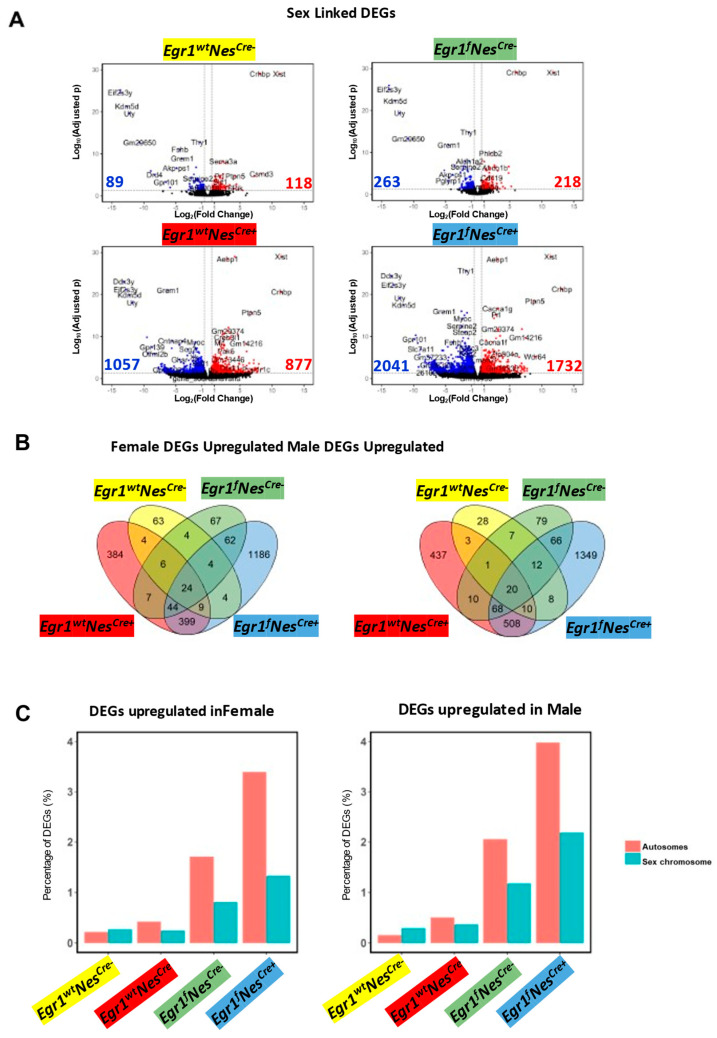
Gene expression changes associated with sex difference. (**A**) Volcano plot for pairwise analysis between two sexes for four genotypes. DEGs were defined as genes with FCs ≥ 1.5 and adjusted *p* values ≤ 0.05. Blue dots indicate downregulated DEGs in males. Red dots indicate upregulated DEGs in males. Grey dots indicate no DEGs. (**B**) Venn diagram showing the overlapped DEGs identified in the pairwise comparisons. Corresponding genotypes are color-coded (blue for *Egr1^f^*Nes^cre+^, green for *Egr1*^f^Nes^cre−^, red for *Egr1*^wt^Nes^cre+^, and yellow for *Egr1*^wt^Nes^cre−^). (**C**) Chromosome distribution of DEGs.

We thus performed pairwise comparisons for the four genotypes separately to identify the genes associated with sex-specific pituitary expression. For the wild-type mice (*Egr1*^wt^Nes^cre−^), a total of 217 genes (99 downregulated and 118 upregulated) were observed in the females (Figure 4A, Supplementary Tables S1–S4). The number of differentially expressed genes (DEGs) involved in sex-related differences increased to 481 in the *Egr1*^f^Nes^Cre−^ mice, 1925 in the *Egr1*^wt^Nes^Cre+^ mice, and 3822 in the *Egr1*^f^Nes^Cre+^ mice. A large number of the DEGs identified in the two sexes were genotype-specific (Figure 4B, Supplementary Tables S1–S4). Only 24 upregulated genes in females and 20 upregulated genes in males were found to be shared by all four genotypes. These shared genes are predominantly from the sex chromosomes and include Ddx3y and Kdm5d on the Y chromosome and Xist and Kdm6a on the X chromosome. For each pairwise comparison, we calculated the percentage of DEGs that were identified in autosomes and sex chromosomes (Figure 4C). Compared with the wild-type controls, the percentage of DEGs in autosomes increased by approximately 3.5 and 4.0 times for the female and male *Egr1*^f^Nes^cre+^ mice, respectively. For all four genotypes, the percentage of upregulated pituitary DEGs on the sex chromosomes in the males was approximately twice as high as that observed in the females. Altogether, the sex-related differences in pituitary gene expression were amplified in the *Egr1*^f^ and Nes^cre+^ mice and involved a thousand genes on autosomes.

### 3.4. Aberrant Gene Expression Associated with Nestin-Cre and Egr1cKO Mice

**Figure 5:**
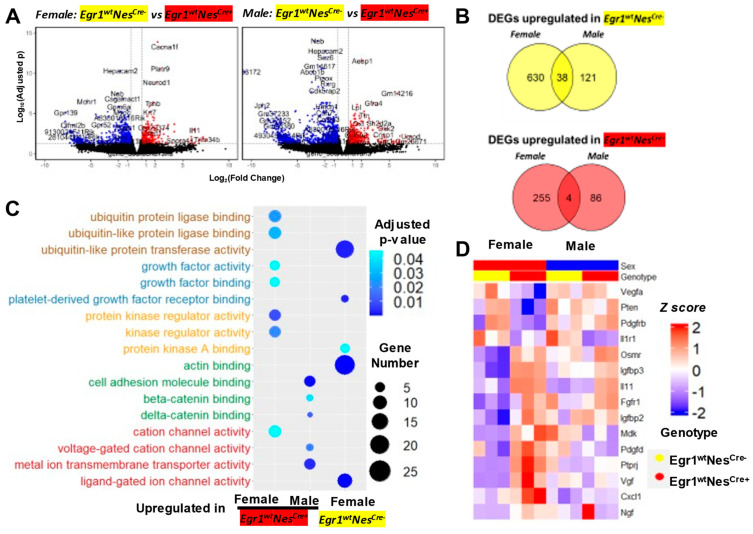
Gene expression changes associated with the Nestin-Cre deriver. (**A**) Volcano plots for pairwise comparisons between female (**left**) and male (**right**) *Egr1*^wt^Nes^cre+^ and *Egr1*^wt^Nes^cre−^ mice. (**B**) Venn diagram showing the overlapped DEGs identified in the pairwise comparisons for the two sexes. (**C**) GO analysis of identified DEGs. (**D**) Gene expression profiles of 15 DEGs were identified in the 3 GO categories associated with growth factors.

The substantial sex-related differences in pituitary gene expression observed in the mice of four different genotypes suggest that the Nestin-Cre driver may have a distinct influence on the development of male and female pituitary tissues. To better understand the effect of the Nestin-Cre driver, we focused on the *Egr1*^wt^Nes^cre+^ and *Egr1*^wt^Nes^cre−^ mice. We still kept the male and female RNA-seq data separate to perform pairwise comparisons (Figure 5A, Supplementary Tables S5 and S6). In the females, 668 genes were downregulated, and the Nestin-Cre driver upregulated 259 genes. Fewer DEGs were found in the males (159 downregulated genes and 90 upregulated genes). Only 38 DEGs upregulated in Egr1wtNescre- and 4 DEGs upregulated in Egr1wtNescre+ pituitary glands were found to be shared by the male and female mice (Figure 5B). Significant differences in the enrichment of GO terms were observed between the female and male DEGs, and none of the GO terms were enriched for genes downregulated by the Nestin-Cre driver in the male pituitary, likely because only a small number of DEGs were detected (Figure 5C).

Five categories of GO terms in biological processes were found to be significantly enriched for DEGs associated with the Nestin-Cre driver. These GO terms are related to growth factors, ubiquitin protein ligase, protein kinase, cell adhesion molecules, and ion channel activity. Interestingly, the genes upregulated and downregulated by the Nestin-Cre driver in females were frequently found in similar GO categories. Although it remains unknown how these genes coordinate with each other in response to the Nestin-Cre driver, the identification of DEGs in the functional pathway related to growth factor may help explain the smaller stature of the Nestin-Cre mice. The expression patterns across 12 samples were examined for the 15 genes associated with the GO terms related to growth factors (Figure 5D). Except for interleukin 1 receptor type 1 (Il1r1), all of the genes were found to be Nestin-Cre-associated DEGs in females, but not in males. Compared with the wild-type controls, the expression of the Il1r1 gene decreased with a fold change of 0.22 in the males and 0.39 in the females. Interleukin 1 (IL-1) stimulates the expression of various kinds of growth factors, including fibroblast growth factor-2 [26] and nerve growth factor [27]. However, the mice without the Il1r1 gene exhibited no overt phenotype, but did exhibit altered innate immune and inflammatory responses [28].

**Figure 6:**
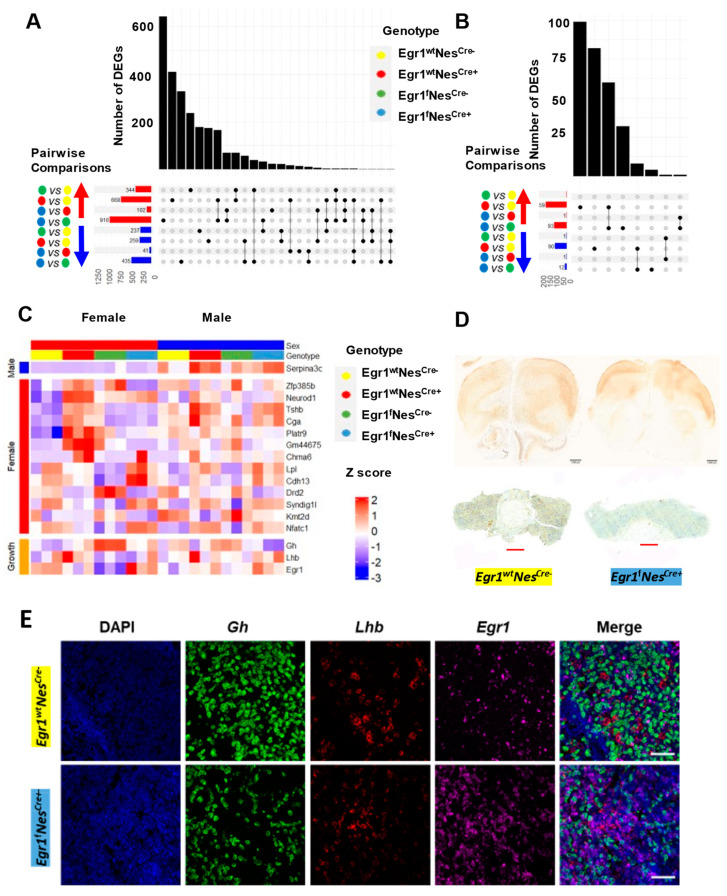
Gene expression changes associated with *Egr1*cKO. Barplots showing the number of intersecting DEGs identified in pairwise comparisons between (**A**) females and (**B**) males. Horizontal bars on the bottom left side of each plot show the numbers of DEGs determined to be upregulated (red arrow) or downregulated (blue arrow). Different intersection combinations of DEGs identified for each pairwise comparison are represented by the dotplot. The vertical barplots show the number of intersecting DEGs in the indicated combinations of pairwise comparisons. (**C**) Gene expression profiles of *Egr1*, growth hormone, and LH_β_ together with DEGs determined to be associated with *Egr1*cKO (1 DEG in males and 11 DEGs in females). (**D**) Immunohistochemistry staining of mouse brain in coronal view (scale bar = 2 mm) and pituitary tissues of *Egr1*cKO mouse and wild-type control. (scale bar = 400 μm). (**E**) RNAscope results for *Egr1*, growth hormone, and LH_β_ expression in the pituitary tissues of *Egr1*cKO and wild-type mice (scale bar = 50 μm). The experiments involving IHC and RNAscope were replicated using biological duplicates.

Since the Nestin-Cre driver led to a large number of genes being differentially expressed in the pituitaries of the two sexes, we performed four kinds of pairwise comparisons to examine the influence of *Egr1*cKO. In the females, more DEGs (916 upregulated and 435 downregulated) were identified between the *Egr1*^f^Nes^cre+^ and *Egr1*^f^Nes^cre−^ mice than the number of DEGs (668 upregulated and 259 downregulated) identified between the *Egr1*^wt^Nes^cre+^ and *Egr1*^wt^Nes^cre−^ mice (Figure 6A). However, in males, an opposite trend in the number of DEGs was observed; more DEGs were identified for the Nestin-Cre driver alone (Figure 6B). Since a pairwise comparison is not sufficient to scrutinize the influence of conditional knockout of the *Egr1* gene, we adopted a mixed-gene model to analyze the pituitary gene expression profiles of the mice of all four genotypes (Supplementary Table S7). Only the *Serpina3c* gene was determined to be associated with *Egr1* loss in the males, while eleven such genes were identified in the females (Figure 6C). We further examined the expression profiles of the *Egr1*, growth hormone, and LH_β_ genes, and they were not differentially expressed across genotypes. Altogether, our results indicate that the conditional knockout of the *Egr1* gene has a more severe impact on pituitary gene expression in females than in males. In addition, in males, the influence of *Egr1*cKO on pituitary gene expression may be overridden by the Nestin-Cre driver.

To validate the presence of EGR1 proteins, immunohistochemistry using an anti-*Egr1* antibody was performed on the brain sections and pituitary tissues obtained from the adult *Egr1*^f^Nes^cre+^ and *Egr1*^wt^Nes^cre−^ mice (Figure 6D). EGR1 proteins were widely distributed across the cerebrum and highly expressed in the hippocampus in the *Egr1*^wt^Nes^cre−^ mice, but they were substantially diminished in the brain sections derived from the *Egr1*^f^Nes^cre+^ mice. However, EGR1 proteins were detected in the pituitary tissues of both the *Egr1*^wt^Nes^cre+^ and *Egr1*^f^Nes^cre−^ mice. This result indicates that the Nestin-Cre driver removes the expression of the *Egr1* gene from neural lineage cells in the brain but fails to knock out the *Egr1* gene in pituitary cells. Lastly, we further confirmed the expression of the *Egr1*, growth hormone, and LH_β_ genes in the pituitary tissues using RNAscope (Figure 6E). For each probe, individual images of the anterior pituitary were taken along with a DAPI control (Figure 6E). Consistent with IHC staining (Figure 6D), *Egr1*, LH_β_, and GH mRNAs were detected in the pituitary glands of both the *Egr1*^f^Nes^cre+^ and *Egr1*^wt^Nes^cre−^ mice. Such findings were observed consistently in both the IHC and RNAscope experiments using biological duplicates.

## 4. Discussion

Nestin-Cre transgenic mice have been widely used to study gene functions in the central nervous system. In this study, we found that the Nestin-Cre driver is able to successfully knock out *Egr1* expression in a large number of brain cells, but that it fails to remove *Egr1* from all three sections of the pituitary. Despite the high expression of Nestin previously found in the pituitary progenitor cells [25], a previous study did not find intensive expression of the Nestin-Cre transgene in the embryonic or adult pituitary [29]. Such inconsistency may be explained by the fact that different reporter mice were used in these studies, and by the unique characteristics of Nestin-Cre expression. During pituitary development, a high level of Nestin expression was observed in POU1F1 (POU domain class 1 transcription factor 1)-positive progenitor cells, but this was followed by a drastic reduction in the postnatal period [24]. The cells with Nestin expression in the postnatal period were terminally differentiated and located in the intermediate and posterior pituitary lobes. However, Nestin-Cre mediated recombination is extremely infrequent in early embryonic progenitor cells, but can reach nearly 100% in neural and glial progenitor cells during perinatal development [30]. In this study, our results concerning *Egr1* mRNA and protein expression profiles have further confirmed the speculation that this specific strain of Nestin-Cre (B6.Cg-*Tg (Nes-cre)1Kln*/J; Jackson Lab, #003771) may not be suitable for pituitary gene knockout.

Despite this limitation, we made several interesting observations. Both *Egr1* loss and the Nestin-Cre driver have been reported to be associated with growth abnormalities in transgenic mice [17,23]. In this study, for mice of four genotypes related to *Egr1*cKO driven by Nestin-Cre, we provided growth curves to monitor their postnatal changes in detail. The differences in growth rate resulting from the Nestin-Cre driver alone became significant as young adulthood approached and were consistent after eight weeks. Compared with the female groups, the *Egr1*^wt^Nes^cre+^ males with the Nestin-Cre driver showed more significant differences from the controls, but no additional weight loss was observed in the *Egr1*^f^Nes^cre+^ males with the combination of Nestin-Cre and *Egr1* knockout in the neuronal lineage cells of the brain. However, *Egr1* knockout led to slightly decreased body weight in the *Egr1*^f^Nes^cre+^ females compared with the *Egr1*^wt^Nes^cre+^ Females. This sex-related difference was further manifested in the gene expression profiles. Considering the significant role of the pituitary in the regulation of growth and development, we explored whether and how its expression profile may be altered in response to the loss of *Egr1*. To our surprise, the *Egr1* gene was indeed removed from the neuronal lineage cells in the brain, but it was still expressed in the pituitary cells. This raises the possibility that the reductions in body size may have been due to the loss of *Egr1* in the neuronal lineage cells. Regardless, we determined in this study that this loss of *Egr1* expression resulted in substantial gene expression changes in the pituitary and affected the activities of growth factors.

In our study, a total of 217 genes were determined to be differentially expressed in the pituitary tissues of male and female wild-type controls. This difference increased approximately two-fold in the *Egr1*^f^Nes^Cre−^ mice, five-fold in the *Egr1*^wt^Nes^Cre+^ mice, and nineteen-fold in the *Egr1*^f^Nes^Cre+^ mice. This result suggests that *Egr1*^f^ and Nes^Cre+^ take on distinct functions, but that together they amplify their impact on the regulation of sex-related pituitary gene expression. The presence of the Nestin-Cre driver alone resulted in 927 differentially expressed genes in the female pituitary, but only 249 genes were affected in the male pituitary tissue. A GO term analysis showed that the pathway associated with growth factor was enriched in the list of differentially expressed genes. This may help explain the growth changes observed in the Nestin-Cre mice. Using a mixed-gene model, we identified a small set of differentially expressed genes associated with the loss of *Egr1* in neuronal lineage cells, but the strong influence of the Nestin-Cre driver may undermine the impact of *Egr1* on pituitary gene expression. Future studies using different Cre lines are highly desired to further understand the roles of *Egr1* in pituitary functions.

## 5. Conclusions

Altogether, our results indicate that the Nestin-Cre driver was able to efficiently remove *Egr1* genes from neuronal lineage cells in the brain, but not from pituitary cells. An RNA-seq analysis revealed that growth factor-related gene pathways were disrupted by the Nestin-Cre driver. In addition, sex differences in growth and gene expression were observed in the *Egr1*cKO and Nestin-Cre mice, and female mice were more sensitive to the presence of Nestin-Cre.

## Data Availability

The RNA-seq datasets generated in this study have been deposited in the NCBI Gene Expression Omnibus (GEO) under accession number GSE108768.

## Supplementary Materials

The following supporting information can be downloaded at: https://www.mdpi.com/article/10.3390/biology12070966/s1, Supplementary Table S1. Full list of DEG sex differences, female vs. male (*Egr1*^wt^Nes^cre−^); Supplementary Table S2. Full list of DEG sex differences, female vs. male (*Egr1*^f^Nes^cre−^); Supplementary Table S3. Full list of DEG sex differences, female vs. male (*Egr1*^wt^Nes^cre+^); Supplementary Table S4. Full list of DEG sex differences, female vs. male (*Egr1*^f^Nes^cre+^); Supplementary Table S5. Full list of DEG differences between female *Egr1*^f^Nes^cre+^ and *Egr1*wtNes^cre+^; Supplementary Table S6. Full list of DEG differences between male *Egr1*^f^Nes^cre+^ and *Egr1*wtNes^cre+;^ Supplementary Table S7. Full list of DEGs derived from mixed-model analysis.

## Abbreviations

GH	Growth hormone
LH	Luteinizing hormone
PRL	Prolactin
TSH	Thyroid-stimulating hormone
FSH	Follicle-stimulating hormone
Egr1	Early growth response 1
*sf1*	Steroidogenic factor 1
*Pitx1*	Pituitary homeobox factor 1
EGR1	Early growth response 1
GnRH	Gonadotropin-releasing hormone
PCA	Principal component analysis
GO	Gene ontology
